# Efficacy and safety of lactulose for the treatment of irritable bowel syndrome

**DOI:** 10.1097/MD.0000000000017295

**Published:** 2019-09-27

**Authors:** Hong-Bin Chen, Xiao-Ying Su

**Affiliations:** a Department of Gastroenterology; b Department of Nursing, Shaanxi Second People's Hospital, Xi’an, China.

**Keywords:** efficacy, irritable bowel syndrome, lactulose, randomized controlled trial, safety

## Abstract

**Background:**

In this study, investigators will evaluate the efficacy and safety of lactulose for the treatment of irritable bowel syndrome (IBS).

**Methods:**

Literature search for relevant studies up to present will be conducted in MEDICINE, EMBASE, Google Scholar, Web of Science, Cochrane Library, Wangfang, Allied and Complementary Medicine Database, Chinese Biomedical Literature Database, and China National Knowledge Infrastructure. The included studies are randomized controlled trials of lactulose in patients with IBS. We will use RevMan 5.3 software using statistical analysis.

**Results:**

This study will provide a high-quality integration of current evidence of lactulose for treating IBS on several aspects including global IBS symptoms, abdominal pain, defecation urgency, stool frequency, stool consistency, quality of life, and adverse events.

**Conclusions:**

This study will provide the evidence for the clinical efficacy and safety of lactulose for the treatment of IBS.

**PROSPERO registration number:**

PROSPERO CRD42019140639.

## 1 Introduction

Irritable bowel syndrome (IBS) is a very common and functional gastrointestinal disease.^[1–3]^ It is diagnosed based on symptoms and is characterized by recurrent abdominal pain and discomfort, excess gas, diarrhea or constipation, and stool pattern.^[4–6]^ It has been estimated that the prevalence of IBS is about 5% to 22% among general population experiencing IBS,^[7]^ and such number is about 5% to 10% in China.^[8–10]^ Its incidence presents a persistently increasing tread.^[11–13]^ Thus, it is very important to treat this disorder effectively. Fortunately, several studies have reported that lactulose has been widely utilized for the treatment of IBS.^[14–18]^ However, its efficacy for IBS is still inconclusive, and no study has been addressed this issue. Therefore, this study will systematically assess the efficacy and safety of lactulose for the treatment of patients with IBS.

## 2 Methods

### 2.1 Study selection criteria

#### 2.1.1 Types of studies

This study will include all randomized controlled trials (RCTs) of lactulose for the treatment of IBS without publication status. However, other studies will be excluded, such as animal studies, case studies, and non-RCTs.

#### 2.1.2 Types of interventions

The experimental treatments must be any forms of lactulose.

The control therapies can be any interventions, except lactulose.

#### 2.1.3 Types of patients

Regardless of any limitations of race, sex, age, and economic status, the patients diagnosed as IBS will be included.

#### 2.1.4 Types of outcome measurements

The outcomes consist of global IBS symptoms; abdominal pain; defecation urgency; stool frequency; stool consistency, as measured by Bristol score; quality of life, as measured by Short Form-36 Health Survey; and adverse events.

### 2.2 Search strategy

The following databases will be searched up to the present: Cochrane Library, MEDICINE, EMBASE, Google Scholar, Web of Science, Wangfang, Allied and Complementary Medicine Database, Chinese Biomedical Literature Database, and China National Knowledge Infrastructure. We will also search other literature records, such as trial registry, dissertations, and conference proceedings. The search strategies designed for Cochrane Library are presented in Table 1. Similar modified search strategies will be applied to the other databases. No language limitation will be imposed.

**Table 1:**
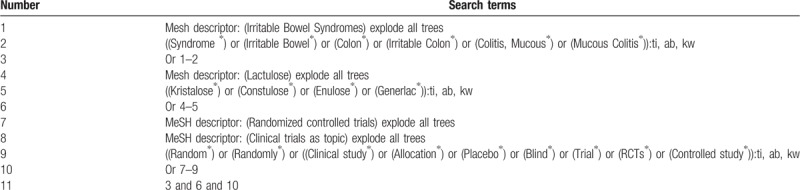
Search strategy details for Cochrane Library.

### 2.3 Data collection and analysis

#### 2.3.1 Study selection

All studies searched by both electronic databases and grey literature. Two investigators will independently scan the titles, abstracts, and records of all the studies to explore more potential studies according to the previous defined eligibility criteria. All disagreements will be solved by a consensus and discussion with the help of a third investigator. The reasons excluding studies will be recorded and presented in the flowchart.

#### 2.3.2 Data extraction

Two investigators will independently extract data and fill the standard data extraction sheet, which will consist of study information, such as first author, publication year, study design, study methods, treatment details, outcomes, safety, and follow-up details. All different opinions between 2 investigators will be solved via consensus and discussion. A third arbiter will be invited to reach an agreement.

#### 2.3.3 Missing data dealing with

In case of missing or unclear data, we will try to contact original authors through obtain sufficient data. Despite such attempts, if we cannot obtain the data, it will be performed based on the intent-to-treat principle.

#### 2.3.4 Risk of bias assessment

Two investigators will assess the risk of bias based on the Cochrane risk of bias tool. This tool includes seven aspects, and each assessment outcome will be showed via 1 of the 3 types: low, unclear, and high risk of bias. All disagreements will be solved by a consensus and discussion between 2 investigators, and if necessary, the third investigator will intervene.

#### 2.3.5 Methods of treatment measurements

Dichotomous data will be assessed via risk ratio and 95% confidence intervals, and continuous data will be expressed through mean difference or standardized mean difference and 95% confidence intervals.

#### 2.3.6 Heterogeneity assessment

We will use *I*^*2*^ statistics to evaluate the heterogeneity. *I*^*2*^ ≤ 50% indicates acceptable heterogeneity, while *I*^*2*^ > 50% means significant heterogeneity.

#### 2.3.7 Assessment of reporting bias

If the analysis consists of more than 10 RCTs, we will conduct funnel plot and Egger regression test to assess the publication bias or small-study effects.^[19,20]^

### 2.4 Data synthesis

RevMan 5.3 software will be utilized to perform statistical analysis. If the heterogeneity is acceptable (*I*^*2*^ ≤ 50%), a fixed-effect model and meta-analysis will be applied. If the heterogeneity is substantial (*I*^*2*^ > 50%), a random-effect model will be performed, and subgroup analysis will be carried out to identify possible reasons for such high heterogeneity. This study will not pool the data if there is still significant heterogeneity after subgroup analysis. Outcome results will be reported as narrative summary.

#### 2.4.1 Subgroup analysis

To explore the potential sources of heterogeneity, we will carry out subgroup analysis according to the different characteristics, interventions, and outcome measurements.

#### 2.4.2 Sensitivity analysis

Sensitivity analysis will be conducted to assess the robustness of pooled outcome results by removing studies with high risk of bias.

## 3 Discussion

Several previous studies have explored the efficacy and safety of lactulose for the treatment of IBS. However, to the best of our knowledge, this study will be the first one to comprehensively assess current available treatments through quantitative methods. The results of this study will provide information on the credibility current evidence and research directions for patients, physicians, and clinical researchers.

### 3.1 Ethics and dissemination

The expected goal is disseminating this study at peer reviewed publication. The ethical approval is not inquired in this study, because no participants’ privacy will not be involved.

## Author contributions

**Conceptualization:** Hong-bin Chen, Xiao-ying Su.

**Data curation:** Hong-bin Chen, Xiao-ying Su.

**Formal analysis:** Hong-bin Chen.

**Funding acquisition:** Xiao-ying Su.

**Investigation:** Xiao-ying Su.

**Methodology:** Hong-bin Chen.

**Project administration:** Xiao-ying Su.

**Resources:** Hong-bin Chen.

**Software:** Hong-bin Chen.

**Supervision:** Xiao-ying Su.

**Validation:** Hong-bin Chen, Xiao-ying Su.

**Visualization:** Hong-bin Chen, Xiao-ying Su.

**Writing – original draft:** Hong-bin Chen, Xiao-ying Su.

**Writing – review & editing:** Hong-bin Chen, Xiao-ying Su.
